# The AMADEUS score is not a sufficient predictor for functional outcome after high tibial osteotomy

**DOI:** 10.1186/s40634-023-00575-2

**Published:** 2023-01-27

**Authors:** Tizian Heinz, Felix Meller, Karsten Sebastian Luetkens, Philip Mark Anderson, Ioannis Stratos, Konstantin Horas, Maximilian Rudert, Stephan Reppenhagen, Manuel Weißenberger

**Affiliations:** 1 Department of Orthopaedic Surgery, University of Wuerzburg, Koenig-Ludwig-Haus, Brettreichstr. 11, 97074 Wuerzburg, Germany; 2 Department of Diagnostic and Interventional Radiology, University Hospital Wuerzburg, Oberduerrbacherstr. 6, 97080 Wuerzburg, Germany

**Keywords:** Cartilage, AMADEUS, KOOS, Knee, High tibial osteotomy, Chondral defect, Osteoarthritis, PROM, Correlation

## Abstract

**Purpose:**

The Area Measurement And Depth Underlying Structures (AMADEUS) classification system has been proposed as a valuable tool for magnetic resonance (MR)-based grading of preoperatively encountered chondral defects of the knee joint. However, the potential relationship of this novel score with clinical data was yet to determine. It was the primary intention of this study to assess the correlative relationship of the AMADEUS with patient reported outcome scores in patients undergoing medial open-wedge high tibial valgus osteotomy (HTO). Furthermore, the arthroscopic ICRS (International Cartilage Repair Society) grade evaluation was tested for correlation with the AMADEUS classification system.

**Methods:**

This retrospective, monocentric study found a total of 70 individuals that were indicated for HTO due to degenerative chondral defects of the medial compartment between 2008 and 2019. A preoperative MR image as well as a pre-osteotomy diagnostic arthroscopy for ICRS grade evaluation was mandatory for all patients. The Knee Osteoarthritis Outcome Score (KOOS) including its five subscale scores (KOOS-ADL, KOOS-QOL, KOOS-Sports, KOOS-Pain, KOOS-Symptoms) was obtained preoperatively and at a mean follow-up of 41.2 ± 26.3 months. Preoperative chondral defects were evaluated using the AMADEUS classification system and the final AMADEUS scores were correlated with the pre- and postoperative KOOS subscale sores. Furthermore, arthroscopic ICRS defect severity was correlated with the AMADEUS classification system.

**Results:**

There was a statistically significant correlation between the AMADEUS BME (bone marrow edema) subscore and the KOOS Symptoms subscore at the preoperative visit (r = 0.25, *p* = 0.04). No statistically significant monotonic association between the AMADEUS total score and the AMADEUS grade with pre- and postoperative KOOS subscale scores were found. Intraoperatively obtained ICRS grade did reveal a moderate correlative relation with the AMADEUS total score and the AMADEUS grade (r = 0.28, *p* = 0.02).

**Conclusions:**

The novel AMADEUS classification system largely lacks correlative capacity with patient reported outcome measures in patients undergoing HTO. The MR tomographic appearance of bone marrow edema is the only parameter predictive of the clinical outcome at the preoperative visit.

## Background

Focal cartilage defects of the knee joint are among others a well-known risk factor predisposing to osteoarthritis (OA) of the whole joint. With the raising socioeconomic burden due to osteoarthritis of the knee joint, surgical treatment modalities addressing the recovery of cartilage defects and thereby hindering the accelerated progression to OA are getting increasingly important [13, 15, 19]. Medial open-wedge high tibial valgus osteotomy (HTO) aims to slow down cartilage deterioration in a varus deformity knee by unloading the medial compartment and thereby restoring knee function [1]. The choice of cartilage repair techniques deeply depends on a thorough evaluation of the cartilage defect severity.

It is the purpose of the recently reported AMADEUS (Area Measurement And Depth Underlying Structures) score to aid in the meticulous defect severity grading. This magnetic resonance (MR) – based classification system allows for a thorough assessment of the cartilage defect area and defect depth including the integrity of the subchondral bone which can be transferred to a total score ranging from zero to 100 (0 = worst score, 100 = best score) [11]. Furthermore, the total AMADEUS score can be transformed to an AMADEUS grade ranging from I to IV (I = best grade, IV = worst grade) for an ICRS (International Cartilage Repair Society) like appearance [11]. Relative association of the AMADEUS classification system with the arthroscopically assessed ICRS classification system has already been demonstrated [7], the association of the AMADEUS with patient-reported outcome measures (PROMs) is still widely unknown. Therefore, this is the first study to investigate the correlative capacity of the AMADEUS classification system with the Knee Osteoarthritis Outcome Score (KOOS) in patients undergoing HTO due to degenerative chondral lesions of the medial compartment. Furthermore, the arthroscopically assessed ICRS grade was to evaluate for its relationship with the MR-based AMADEUS system. A weak correlative association between the AMADEUS grading system and the KOOS was hypothesized.

## Materials and methods

### Study population and design

This retrospective study was approved by the local ethics committee and the requirement for informed consent was waived. A total of 70 patients were indicated for HTO at a single university orthopedic center from 2008 to 2019 and data were retrospectively analyzed by means of digitally archived medical records. Inclusion criteria for this study were defined as follows: (1) enduring knee pain predominantly of the medial compartment of the knee, (2) varus deformity of the tibial plateau of the affecting limb, (3) preserved and intact lateral and retropatellar knee compartment, (4) preoperatively performed MR exam of the knee of sufficient quality for evaluation of the chondral integrity, (5) either radiographically (MRI) or arthroscopically diagnosed focal cartilage defect of the medial compartment. Patients with early osteoarthritic changes of the medial compartment rather than a focal cartilage defect were not found eligible for this study. At the beginning of the study, a sample size calculation was performed based on the hypothesis of a weak correlation between the AMADEUS and the KOOS.

### Surgical technique

All patients were obligated to obtain a full-length weight bearing radiograph of the lower limb before surgery. Correction deformity was planned according to the radiographs and the correction angle of the opening wedge hinge was calculated. Target parameters for the correction osteotomy were defined as follows: 1) Intersection of the weightbearing line at 62.5% of the mediolateral tibial plateau width which roughly corresponds to the lateral tibial spine in the coronal plane; 2) a mechanical femorotibial angle of 3 to 5 degrees of valgus were desired; 3) Relocating the weight bearing axis through the Fujisawa point (i.e. 30% to 40% lateral to the midpoint) [6]. Surgical-wise, a medial approach was used and a biplanar L-shaped medial osteotomy was subsequently performed. The posteromedial site was gradually opened until the desired correction angle was achieved. The correction angle was checked intraoperatively using fluoroscopy. Afterwards, the osteotomy site was secured using plate fixation (TomoFix, Synthes, Solothan, Switzerland). The postoperative protocol encompassed a six-week period of non-weightbearing ambulation accompanied by passive motion exercises. A braced hinge allowing the knee-flexion up to 90 degrees was further applied for 6 weeks. After radiographic controls were obtained 6 weeks postoperatively progressive weightbearing was commenced and knee flexion was no longer limited.

### Patient reported outcome measures (PROMs)

Patient reported outcome measures were evaluated using the KOOS including its five sub-divisions (KOOS-Pain, KOOS-Symptoms, KOOS-ADL, KOOS-QOL and KOOS-Sports) and established guidelines for final scoring were used [3]. For this study, all patients were asked to fill in the KOOS questionnaire the day before surgery and at specific re-visits postoperatively.

### AMADEUS grading

**Fig. 1:**
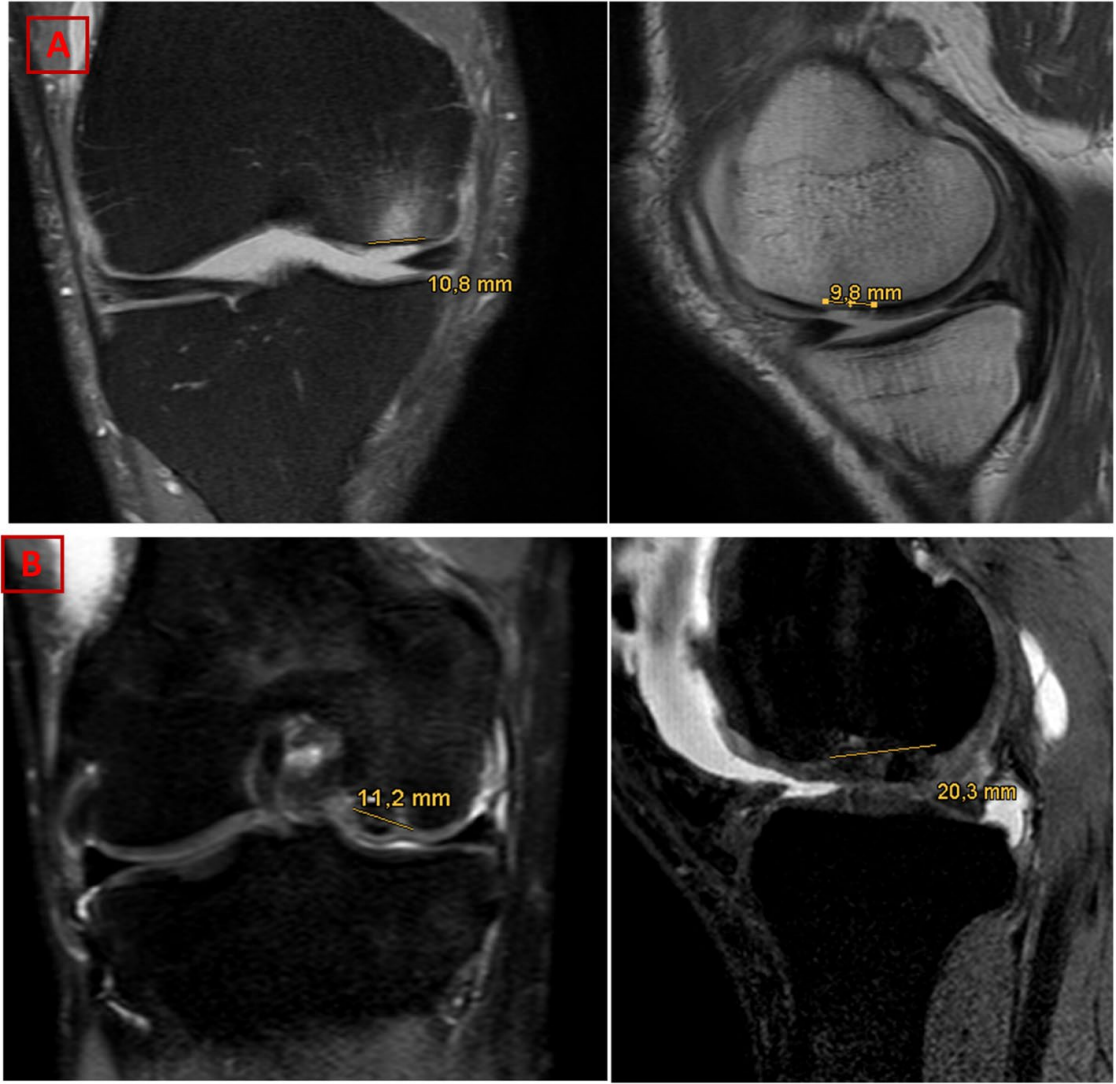
Example MR images for illustration of chondral defect severity grading according to the AMADEUS protocol. **A** Proton density fat saturated (coronal view) and T2-weighted (sagittal view) MRI images. Example of a patient with a full chondral defect but with an intact subchondral layer. The defect area was measured as 1.06 cm^2^. A bone marrow edema is clearly visible in the proton density fat weighted images (cornal view). This corresponds to a total AMADEUS score of 60 points: First digit “Area”: 30 points (> 1 cm^2^ to ≤ 2 cm^2^), second digit “Depth”: 0 points (full thickness), third digit “Underlying Structures”: 30 points (no bony defect), fourth digit “Bone Marrow Edema”: 0 points (present bone marrow edema). **B** Proton density fat saturated (coronal view) and T2-weighted fast spine echo (sagittal view) images. Example of a patient with a full chondral defect and concomitant lesion of the subchondral layer. The defect area was measured as 2.27 cm^2^. A bone marrow edema was not visible. This corresponds to a total AMADEUS of 30 points: First digit “Area”: 20 points (> 2 cm^2^ to ≤ 4 cm^2^), second digit “Depth”: 0 points (full thickness), third digit “Underlying Structures”: 0 points (Bony defect > 5 mm), fourth digit “Bone Marrow Edema”: 10 points (no present bone marrow edema)

The routinely obtained preoperative MR-scans of the affected knee were analyzed by one experienced and in musculoskeletal imaging trained radiologist who was otherwise not involved in the clinical setting of this study, as well as by one physician in residency training for orthopedic surgery. Chondral defects of the medial compartment were evaluated according to the AMADEUS protocol recently published by Jungmann et al. [11]. Sagittal and coronal images of a cartilage sensitive sequence (i.e. MR images of an intermediate-weighted (IM) or T2-weighted fast spin echo (FSE) sequence or a proton-density (PD) weighted sequence in combination with an T2-weighted FSE in at least two planes) were used for evaluation of the total defect size area. Furthermore, defect depth of the chondral defect was graded into four increments (no defect, signal alteration, partial thickness, and full thickness) and the integrity of the subchondral bone was evaluated separately with three increments (no bony defect, bony defect ≤ 5 mm in depth and bony defect > 5 mm in depth). Additionally, the presence of a surrounding bone marrow edema (BME) was also noted. Based on the subscores, a total AMADEUS score ranging from zero (severe cartilage defect) to 100 (no osteochondral defect) was formed. The AMADEUS total score was then transferred to an AMADEUS grade ranging from I (minimal defect severity) to IV (highest defect severity) according to the AMADEUS protocol recently published [11]. Example images for defect severity grading are given in Fig. 1.

### Statistics

Statistical analysis was performed using SPSS statistical software (SPSS, Chicago, IL, USA, Version 27). A *p*-value of 0.05 was set as level of significance. Normal distribution was tested using the Kolmogorov-Smirnov test and Shapiro-Wilk test. Distribution und quantities were assessed for categorical variables. For numeric data, mean values and standard deviations were calculated. Differences between pre- and postoperative values in the PROMs scores were assessed using a paired T-Test or one-way ANOVA analysis with a post-hoc Bonferroni Test. In case of non-parametric data, the Mann-Whitney U or Kruskal-Wallis-Test were used instead. Correlative relationship between the different variables was calculated using to the non-parametric Spearman rank correlation coefficient (Spearman R). The strength of the association was evaluated according to the suggestions of Chan et al. [4]. Interrater reliability was calculated using the cohens kappa coefficient. A sample size calculation was performed before the study, assuming a weak correlation (r = 0.3) between the AMADEUS and KOOS scores aiming at statistical power of 0.80. Based on these parameters, a minimal number of 67 patients were needed.

## Results

A total of 70 patients were included in this study with a strong prevalence of male patients (64 male patients vs. six female patients). Descriptive data of the patient cohort are depicted in Table 1.

**Table 1:** Descriptive data of the patient cohort

Variable	Total number or mean ± SD
Gender	
Male	64
Female	6
Age at surgery (years)	44.07 ± 9.06
BMI (kg / m^2^)	27.13 ± 3.36
Follow-up (months)	50.08 ± 26.52
Defect area (cm^2^)	1.14 ± 1.07
ICRS	2.90 ± 0.60
Kellgren-Lawrence	2.41 ± 0.94
LDFA_preoperative_ (degrees °)	88.16 ± 4.98
MPTA_preoperative_ (degrees °)	86.38 ± 4.47
WBL_preoperative_ crossing on mediolateral tibial plateau width (%)	25.69% ± 22.83%
LDFA_postoperative_ (degrees °)	88.09 ± 1.87
MPTA_postoperative_ (degrees °)	91.54 ± 2.95
WBL_postoperative_ crossing on mediolateral tibial plateau width (%)	54.95% ± 11.47%
Previous meniscectomy (partial)	27 (38.57%)

**Fig. 2:**
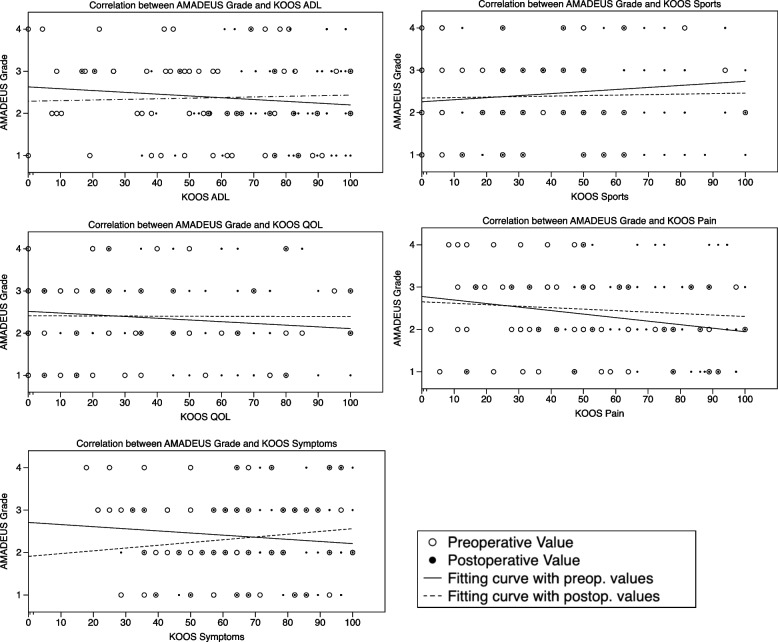
Correlation between the AMADEUS grade and clinical data according to KOOS subscale scores

**Fig. 3:**
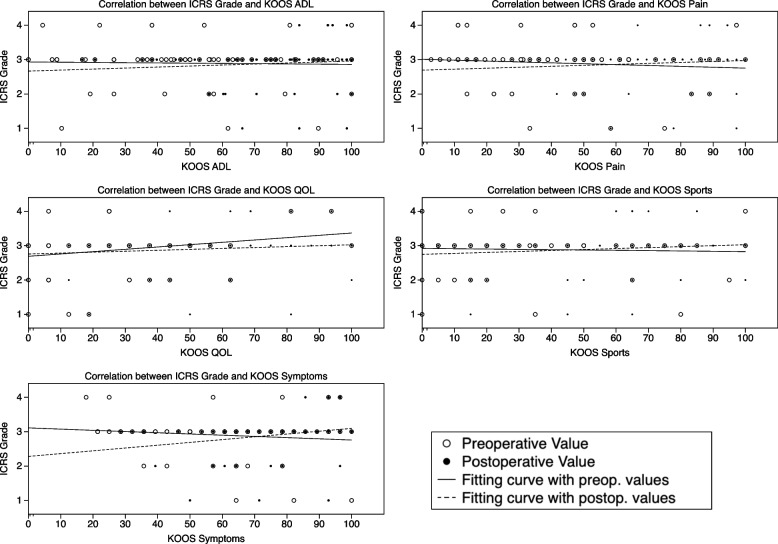
Correlation between the ICRS Grade and clinical data according to the KOOS subscale scores

Regarding the AMADEUS subscores, there was a statistically significant correlation between the AMADEUS BME (bone marrow edema) subscore and the KOOS-Symptoms subscore at the preoperative visit (Table 2, Fig. 2). No statistically significant monotonic association between the AMADEUS total score and the AMADEUS grade with pre- and postoperative KOOS subscale scores were found. Intraoperatively obtained ICRS grade did reveal a moderate correlative relation with the AMADEUS total score and the AMADEUS grade (Table 3). However, the ICRS grade did not reveal a correlative capacity with the pre- and postoperative KOOS subscales scores (Fig. 3).

**Table 2:** Correlation of the AMADEUS total score and AMADEUS subscores with the KOOS subscale scores (Sp. R = Spearman R)

		AMADEUS Total		AMADEUS Defect Area		AMADEUS Defect Depth		AMADEUS Underlying Structure		AMADEUS BME		AMADEUS Grade	
		Sp. R	p	Sp. R	p	Sp. R	p	Sp. R	p	Sp. R	p	Sp. R	p
**Preoperative**	**KOOS ADL**	0.18	0.15	−0.08	0.71	0.01	0.91	− 0.05	0.67	− 0.01	0.93	− 0.1	0.42
	**KOOS QOL**	− 0,11	0.40	−0.15	0.22	−0.10	0.39	−0.08	0.54	−0.09	0.48	0.13	0.31
	**KOOS Symptoms**	0.40	0.75	−0.04	0.75	−0.06	0.63	−0.06	0.65	−0.25	0.04*	−0.11	0.40
	**KOOS Pain**	0.17	0.17	−0.08	0.74	0.04	0.74	0.02	0.89	−0.03	0.83	−0.18	0.15
	**KOOS Sports**	0.25	0.40	−0.08	0.73	−0.02	0.88	−0.09	0.47	−0.06	0.61	−0.12	0.33
**Postoperative**	**KOOS ADL**	0.01	0.96	0.00	0.97	−0.16	0.19	−0.07	0.56	0.01	0.95	0.06	0.61
	**KOOS QOL**	−0.84	0.50	−0.11	0.37	−0.15	0.24	−0.00	0.98	−0.08	0.51	0.05	0.70
	**KOOS Symptoms**	−0.13	0.20	−0.07	0.40	−0.22	0.08	−0.12	0.13	−0.20	0.11	0.10	0.40
	**KOOS Pain**	0.05	0.67	−0.06	0.23	−0.15	0.22	−0.15	0.23	0.07	0.60	−0.11	0.39
	**KOOS Sports**	0.06	0.64	−0.01	0.91	−0.16	0.21	−0.13	0.31	−0.07	0.60	0.02	0.70

**Table 3:** Correlation analysis of the arthroscopically assessed ICRS grade with the AMADEUS total score and AMADEUS grade of both raters

		AMADEUS total Score (Rater 1)	AMADEUS total Score (Rater 2)	AMADEUS Grade (Rater 1)	AMADEUS Grade (Rater 2)
**ICRS Grade**	**Pearson r**	- 0.25	- 0.28	0.28	0.23
	***p*-value**	0.04	0.02	0.02	0.04

**Fig. 4:**
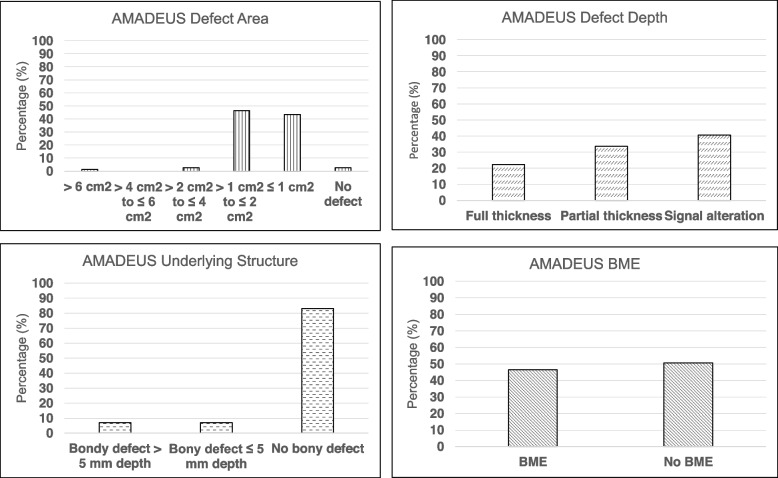
Distribution of the various AMADEUS subscores

The mean AMADEUS total score including the AMADEUS subscores as well as the pre- and postoperative KOOS subscores are depicted in Table 4. Figure 4 shows the distribution of the four AMADEUS subscores within the patient collective. Furthermore, mean KOOS subscore values did not differ significantly between distinct AMADEUS grades at the pre- and postoperative visit (Table 5). Patients with concomitant BME surrounding the cartilage defect area had a significantly lower KOOS-Symptoms subscore at the preoperative visit (*p* = 0.04). This finding was not present for the postoperative visit.

**Table 4:** Mean AMADEUS total score, mean AMADEUS subscores as well as pre- and postoperative KOOS scores of the patient cohort

	AMADEUS Defect Area	AMADEUS Defect Depth	AMADEUS Underlying Structure	AMADEUS BME	AMADEUS Total	KOOS ADL	KOOS QOL	KOOS Sports	KOOS Pain	KOOS Symptoms	
**Mean**	31.81	9.78	27.10	5.22	52.50	**52.88**^a^	**30.50**^a^	**30.05**^a^	**45.16**^a^	**61.46**^a^	**Preoperative**
**SD**	5.21	5.84	8.06	5.03	16.91	28.19	22.26	31.04	25.16	21.76	
**Minimum**	0	0	0	0	10	0	0	0	2.78	17.86	
**Maximum**	40	15	30	10	85	100	100	100	100	100	
**Mean**						**77.98**^a^	**51.30**^a^	**51.38**^a^	**71.99**^a^	**75.59**^a^	**Postoperative**
**SD**						19.99	26.08	28.37	21.26	18.79	
**Minimum**						16.67	12.50	0	13.89	28.57	
**Maximum**						100	100	100	100	100	

^a^Asterisks indicate a statistically significant increase from the mean pre- to postoperative KOOS value of a distinct KOOS subscore

**Table 5:** Pre- and postoperative mean KOOS subscores in respect to the different AMADEUS grades of the chondral lesion. No significant differences were found within a KOOS subscale for the different AMADEUS grades

AMA-DEUS Grade	KOOS ADL preop.	KOOS ADL postop.	KOOS QOL preop.	KOOS QOL postop.	KOOS Symptoms preop.	KOOS Symptoms postop.	KOOS Pain preop.	KOOS Pain postop.	KOOS Sports preop.	KOOS Sports postop.
	Mean (SD)	Mean (SD)	Mean (SD)	Mean (SD)	Mean (SD)	Mean (SD)	Mean (SD)	Mean (SD)	Mean (SD)	Mean (SD)
**1**	57.13 ± 27. 29	78.97 ± 20.12	25.00 ± 21.35	52.88 ± 29.27	62.64 ± 20.34	74.45 ± 19.17	49.20 ± 27.26	77.46 ± 23.45	31.92 ± 29.62	56.54 ± 29.54
**2**	54.23 ± 29.03	76.63 ± 17.55	31.81 ± 22.73	50.00 ± 28.87	65.91 ± 18.63	74.12 ± 21.31	49.24 ± 26.24	71.59 ± 21.30	37.20 ± 34.29	48.33 ± 28.83
**3**	50.71 ± 28.03	75.89 ± 27.48	29.83 ± 20.95	48.30 ± 21.75	57.63 ± 23.65	73.21 ± 18.07	43.94 ± 24.99	67.05 ± 21.98	22.38 ± 30.68	46.67 ± 30.55
**4**	46.15 ± 31.57	83.37 ± 15.01	36.11 ± 28.26	59.03 ± 28.14	58.33 ± 28.23	86.11 ± 12.81	30.25 ± 17.04	76.23 ± 17.35	26.11 ± 26.90	60.00 ± 21.65
***p*-value**	0.80	0.82	0.83	0.88	0.78	0.51	0.33	0.64	0.60	0.66

The mean chondral defect sizes of the medial compartment are depicted in Table 6. 74% of the chondral lesions were located at the medial femoral condyle in the central weight-bearing area whereas 26% of chondral defects were found at the medial tibial plateau. Interestingly, the majority of defects was small in total diameter (90.2% below 2 cm^2^) but with a relatively deep extension into the chondral layers (56.3% full or partial thickness defects).

**Table 6:** Chondral defect area by mean, standard deviation, minimum and maximum

	Coronal diameter (cm)	Sagittal diameter (cm)	Defect area (cm^2^)
Mean	1.08	1.05	1.17
SD	0.88	0.37	1.07
Minimum	0.39	0.15	0.20
Maximum	7.90	2.01	8.85

Regarding the interrater reliability of the AMADEUS total score, a fair agreement was found between both raters (k = 0.23, *p* = 0.00).

## Discussion

As a main result of this study, a moderate correlative relation could be demonstrated between the AMADEUS BME subscore and the KOOS-Symptoms subscale at the preoperative visit. However, there was no correlative capacity of the AMADEUS total score or the AMADEUS grade with the KOOS subscale scores. Moreover, the AMADEUS BME subscore did not reveal correlative association with the KOOS-Symptoms and the remaining KOOS subscales at the postoperative visit.

These findings suggest that the MR based evaluation of chondral defect severity as assessed with the AMADEUS is not able to fully reflect the clinical condition in terms of function and pain. This finding is supported by a recent study of Runer et al. who also could not demonstrate a correlative capacity of the AMADEUS classification system with the knee specific IKDC questionnaire at the preoperative visit [23]. However, the only radiographic feature of the AMADEUS that is linked to the clinical condition of knee is the presence of a BME surrounding the cartilage defect. In this study, the presence of a BME was associated with a lower KOOS Symptoms subscale score at the preoperative visit and subanalysis revealed a statistically significant difference in the KOOS-Symptoms subscale score between patients with and without BME. Furthermore, the presence of BME in addition to cartilage defects has been reported as a frequent side effect with an incidence of 55% to 83% [12, 22]. Nevertheless, its role and influence on the functional outcome before and after cartilage repair surgery remains controversial. Niemeyer et al. indicated worse functional outcome in terms of the IKDC score before and after autologous chondrocyte implantation (ACI) in cases with BME [17]. Contrary, Niethammer et al. was not able to demonstrate inferior results in patients with concomitant BME following ACI [18]. Notably, Yang et al. found a nearly significant correlation between the presence of BME and the preoperative functional outcome scores in patients undergoing HTO [25]. Regarding the postoperative visit, the same authors did not find a significant difference in the functional outcome scores concluding that a preoperative BME may not influence the postoperative outcome following HTO [25]. This finding goes along with the results of the present study as there was no difference in the functional outcome scores at the postoperative visit following HTO.

As the second important finding of this study, there was a moderate correlative relation between the MR based AMADEUS total score and AMADEUS grade with the arthroscopically assessed ICRS grade of the cartilage defect. This result is well in line with recently published literature [7]. Especially with current treatment guidelines being based on the ICRS classification system, the AMADEUS classification system yields the potential for a non-invasive diagnostic tool of therapeutic relevance.

The relationship between MR based radiographic findings and its translation into clinical outcome in patients with knee related problems has been topic of ongoing interest from the year one [2, 9, 14]. So far, several studies have been conducted to evaluate a potential correlation of radiographic MRI data with the clinical outcome at the postoperative stage after surgical cartilage repair techniques have been applied [5, 8, 10, 16, 20]. While most of these studies show a relatively high inconsistency between radiographic and clinical data, less is known for a potential correlative relationship at the preoperative visit. Runer et al. investigated the correlation of the novel AMADEUS classification system with various clinical outcome scores including the International Knee Documentation Committee (IKDC), Short-Form-12 (SF-12) and the Core Outcome Measures Index (COMI) in a patient cohort undergoing autologous chondrocyte implantation (ACI) [23]. As a result, they could demonstrate only a weak correlative capacity of the AMADEUS grading with the preoperatively evaluated COMI score [23]. This finding requires further scientific acknowledgement, as it is unusual for a non-knee-specific patient assessed questionnaire to be more sensitive and concurrent with radiographic data than established knee specific questionnaires like the IKDC and KOOS. To our knowledge, the KOOS has not yet put to test for a correlative relationship with the AMADEUS classification system, which seems inevitable, as the KOOS is another highly validated and often used knee specific questionnaire. Tanner et al. analyzed eleven different knee specific self-assessed questionnaires and concluded that the IKDC and KOOS constitute the evaluation tools that are most important and useful to patients with knee related problems [24].

By selecting patients with mainly degenerative chondral defects due to medial overload of the knee joint, the KOOS seemed most appropriate for the patient cohort of this study, as the KOOS has been validated both for osteochondral defects and osteoarthritis (OA) of the knee joint [3, 21]. It seems uncontroversial that the relationship between clinical and radiographic data is remarkedly influenced by the PROMs chosen for a specific subset of patients. A PROM that may demonstrate a weak sensitivity and specificity or may not even yield validation for the subset of patients considered in a study, will negatively influence a potential correlative association.

This study has several limitations and strengths. There was only one study cohort considered for this study. Diversifying the patient population in terms of the applied surgical procedure would have added additional strength to this study. The same holds true for the evaluated PROM. Analysis of various self-assessed generic and knee-specific questionnaires would have amplified the strength of this study. Unfortunately, the native AMADEUS protocol does not regard the size of the adjacent BME which would have enhanced the study quality.

Yet this is the first study investigating the relationship of the novel AMADEUS classification system with the knee specific KOOS questionnaire. Furthermore, this is the first study considering the AMADEUS for patients undergoing HTO. By inclusion of a total of 70 patients, the number of included individuals seemed to be sufficient when compared to similar studies regarding this topic [11, 23].

## Conclusion

The novel AMADEUS classification system largely lacks correlative capacity with patient reported outcome measures in patients undergoing HTO. The MR tomographic appearance of bone marrow edema is the only parameter predictive of the clinical outcome at the preoperative visit.

## Data Availability

The datasets used and/or analyzed during the current study are available from the corresponding author on reasonable request.

## Abbreviations

ACI: Autologous Chondrocyte Implantation
AMADEUS: Area Measurement And Depth and Underlying Structure
COMI: Core Outcome Measures Index
HTO: High tibial valgus osteotomy
ICRS: International Cartilage Repair Society
IKDC: International Knee Documentation Committee
KOOS: Knee Osteoarthritis Outcome Score
MRI: Magnetic resonance imaging
OA: Osteoarthritis
PROM: Patient reported outcome measure
